# Blind RSSD-Based Indoor Localization with Confidence Calibration and Energy Control

**DOI:** 10.3390/s16060788

**Published:** 2016-05-31

**Authors:** Tengyue Zou, Shouying Lin, Shuyuan Li

**Affiliations:** College of Mechanical and Electronic Engineering, Fujian Agriculture and Forestry University, Fuzhou 350002, China; linshouying@fafu.edu.cn (S.Lin); lsyfafu@163.com (S.Li)

**Keywords:** wireless sensor network, indoor localization, back propagation, unscented Kalman filter, energy control

## Abstract

Indoor localization based on wireless sensor networks (WSNs) is an important field of research with numerous applications, such as elderly care, miner security, and smart buildings. In this paper, we present a localization method based on the received signal strength difference (RSSD) to determine a target on a map with unknown transmission information. To increase the accuracy of localization, we propose a confidence value for each anchor node to indicate its credibility for participating in the estimation. An automatic calibration device is designed to help acquire the values. The acceleration sensor and unscented Kalman filter (UKF) are also introduced to reduce the influence of measuring noise in the application. Energy control is another key point in WSN systems and may prolong the lifetime of the system. Thus, a quadtree structure is constructed to describe the region correlation between neighboring areas, and the unnecessary anchor nodes can be detected and set to sleep to save energy. The localization system is implemented on real-time Texas Instruments CC2430 and CC2431 embedded platforms, and the experimental results indicate that these mechanisms achieve a high accuracy and low energy cost.

## 1. Introduction

Indoor localization is an important field of research with numerous applications in modern daily life [1,2,3], such as elderly care [4], miner security [5] and robot guidance [6]. The Global Positioning System (GPS) [7] is a satellite navigation system that provides successful outdoor geospatial positioning service globally. However, it is not feasible for deployment in indoor environments owing to its requirement for a direct view from several satellites [8]. Consequently, the development of non-GPS-based solutions for indoor applications based on existing hardware has gained significant attention in recent years [9,10,11]. The wireless sensor network (WSN) is one suitable platform for this application [12]. The task of the WSN for localization is to determine the distance between a moving target and anchor nodes with known positions on a map based on the signal strength or transmission time [13].

The majority of WSN localization systems estimate the distance using the time of arrival (TOA) [14], time difference of arrival (TDOA) [15], angle of arrival (AOA) [16] or a combination of these methods. These methods require precise synchronization, and very accurate clocks are necessary, which may increase the cost and complexity of implementation [17]. Because the propagation speeds of infrared (IR) and radio frequency (RF) in free air are approximately 3 × 10^8^ m/s, it is difficult to achieve the 10 ns resolution required to measure a distance of 1 m. Thus, acoustic and ultrasonic signals with a speed of 343 m/s in dry air are often used in TOA [18]. Moreover, the target should use a motion device to adjust the angle of the ultrasonic sensor to receive a signal from various directions. Another TDOA method requires more than one transmission technique to acquire the time differences for computation, which requires more complex hardware design [12].

Energy-based techniques, such as the received signal strength (RSS) [19] and received signal strength difference (RSSD) [20], have also been adopted without the use of additional equipment. Energy-based methods have low energy costs and are easy to implement. Thus, it is easier to achieve a balance between energy cost and accuracy. Because an RSSD method requires no prior knowledge of the target, it is suitable for the localization of so-called blind nodes (nodes with unknown transmission parameters) and it is also the main research point in this paper. The position estimation in energy-based algorithms can be divided into two categories: maximum likelihood (ML) and least squares (LS). The ML is asymptotically optimal when the noise statistics are known [21], but it requires high computational resources and memory usage owing to the highly nonconvex function. LS-based estimators have been developed to overcome this problem by transforming the original nonlinear equations into linear equations [22]. Energy-based localization also requires the help of anchor nodes (nodes with known locations) in the system. Owing to the shadowing effects or obstacles, the RSS information provided by anchor nodes involves different levels of noise. The noise influences the accuracy of the estimation in the next step. To increase the accuracy, the anchor nodes with different levels of noise are viewed as different types of reference nodes in the estimation. In this work, a confidence value is assigned to each anchor node to indicate its accuracy for participating in the estimation.

Energy control is another important issue in WSN applications without a fixed supplement of electricity [23,24,25]. The majority of energy control procedures are based on task scheduling [26,27], topology optimization [28,29] and smart clustering [30,31] but do not focus on reducing energy consumption by unnecessary nodes. Several anchor nodes are necessary in current position estimation, whereas others can be set to sleep to save energy. The selection of sleeping nodes is an optimization problem in the system, and the relevant anchor nodes that may be used at the next time point should be awake for preparation. A quadtree structure is introduced in this work to describe the correlation between areas and to recognize the unnecessary nodes.

The contributions of this paper are as follows: (1) a confidence value is proposed for each anchor node to indicate its accuracy for RSSD localization. The mechanism is used to assist in the selection of anchor nodes for position estimation; (2) an error back propagation mechanism is introduced to determine and maintain the table of confidence values. Furthermore, an automatic calibration device is designed to help the learning procedure; (3) the acceleration of the target is sampled to estimate the position during fast movement, and an unscented Kalman filter (UKF) is introduced to reduce the influence of measurement noise; (4) a quadtree structure is introduced to describe the region correlation of the area, and the anchor nodes that are unnecessary are set to sleep to save energy. Thus, the lifetime of the entire localization system can be prolonged.

The remainder of this paper is organized as follows: Section 2 presents the RSSD-based localization model. In Section 3, a confidence value for each anchor node is built and calibrated by the error back propagation method. An automatic calibration device is also presented in this section. Section 4 describes the moving estimation algorithm for the target using the UKF. In Section 5, a quadtree structure is constructed to determine the unnecessary anchor nodes for saving energy, and the metric for the selection of suitable anchor nodes involved in the estimation is introduced. Experiments are performed on real-time embedded hardware implemented on the Texas Instruments (Dallas, TX, USA) CC2430 and CC2431 WSN platforms, and the results are presented in Section 6. Finally, the conclusions are provided in Section 7.

## 2. RSSD-Based Localization Method

RSSD is a popular geolocation method applied in local network systems and is less complex than TOA and TDOA which should be with the additional measuring equipment. Moreover, RSSD requires no prior knowledge of target for estimation [20], such as the transmitted signal power, path loss exponent and carrier frequency, and it is welcomed by blind node without known transmission parameters.

### 2.1. Empirical Path Loss Models

Empirical path loss models are often used to estimate the received signal strength, which can be viewed as a function of parameters, such as the propagation distance, *d*, transmission antenna height, *a_t_*, receiving antenna height, *a_r_*, and carrier frequency of the transmitted signal, *f* [32]. Although there are several different formulas, the dependence of the RSS is generally expressed as a power law, as shown in Equation (1).

(1) $$P_{r}=\frac{C}{d^{\gamma }}P_{t}$$

where *P_t_* represents the transmitted signal power, *C* = *C*(*a_t_*, *a_r_*, *f*) is a positive constant that depends on *a_t_*, *a_r_*, and *f* and *γ* ≥ 2 is a positive constant, called the path loss exponent. On a logarithmic scale, Equation (1) can be reformulated as

(2) $$\begin{array}{l} \Gamma =10\log_{10}(P_{r})=10\log_{10}(CP_{t})-10\gamma \log_{10}(d)\\ =E-10\gamma \log_{10}(d) \end{array}$$

where *E = E(a_t_, a_r_, f, P_t_)* = 10log_10_(CP_t_). If *a_t_, a_r_, f* and *P_t_* are fixed, *E* is constant for all receivers in a system.

### 2.2. Received Signal Strength Difference

Because of their simplicity, RSS methods have been widely used in indoor localization applications under imprecise environments. However, some RSS methods have the requirements on acquiring prior knowledge before estimation, e.g., the transmitted signal power *P_t_*. For uncooperative emitters, the parameters are difficult to obtain, and the values fluctuate among the same type of emitters. Under these situations, the difference in the received signal strength between two emitters is considered to be a new metric for these applications.

Consider an RF emitter at *K*(*x*, *y*) and two receivers at *R*_1_ = (*x*_1_, *y*_1_) and *R*_2_ = (*x*_2_, *y*_2_). Define the distances from *R*_1_ and *R*_2_ to *K* as *d*_1_ and *d*_2_, respectively. Then, the distance from *R*_1_ to *R*_2_ can be represented as *d*_12_ by Equation (3).

(3) $$d_{12}=\sqrt{{\left(x_{1}-x_{2}\right)}^{2}+{\left(y_{1}-y_{2}\right)}^{2}}$$

Denote Γ_1_ and Γ_2_ as the RSS (in dBm) at *R*_1_ and *R*_2_, respectively, and define Γ_12_ = Γ_1_ − Γ_2_. Thus, according to Equation (2), Γ_12_ can be calculated as Equation (4).

(4) $$\Gamma_{12}=10\gamma \log_{10}(d_{2}/d_{1})$$

where the constant *E* cancels out and does not need to be acquired. Then, set *θ*_12_ as shown in Equation (5), and Equation (4) can be rewritten as Equation (6).

(5) $$\theta_{12}=10^{\frac{\Gamma_{12}}{5\gamma }}$$

(6) $${\left(x-x_{2}\right)}^{2}+{\left(y-y_{2}\right)}^{2}=\theta_{12}[{\left(x-x_{1}\right)}^{2}+{\left(y-y_{1}\right)}^{2}]$$

If *θ*_12_ ≠ 1, Equation (6) can be rewritten as Equation (7), which represents a circle with radius $\sqrt{\theta_{12}}d_{12}/\left|1-\theta_{12}\right|$ and center located on point *O*_12_, as shown in Equation (8).

(7) $${\left[x-\frac{x_{2}-\theta_{12}x_{1}}{1-\theta_{12}}\right]}^{2}+{\left[y-\frac{y_{2}-\theta_{12}y_{1}}{1-\theta_{12}}\right]}^{2}=\frac{\theta_{12}d_{12}^{2}}{{\left(1-\theta_{12}\right)}^{2}}$$

(8) $$O_{12}=\left[\frac{x_{2}-\theta_{12}x_{1}}{1-\theta_{12}},\frac{y_{2}-\theta_{12}y_{1}}{1-\theta_{12}}\right]$$

If *θ*_12_ = 1, Equation (6) degenerates to the linear Equation (9).

(9) $$(x_{1}-x_{2})x+(y_{1}-y_{2})y=\frac{x_{1}^{2}+y_{1}^{2}-x_{2}^{2}-y_{2}^{2}}{2}$$

which denotes the perpendicular bisector of the line segment passing through *R*_1_ and *R*_2_. Owing to the same consideration of straight lines and circles under the stereographic projection in mathematics, the straight line described in Equation (9) can also be treated as a special circle.

**Figure 1:**
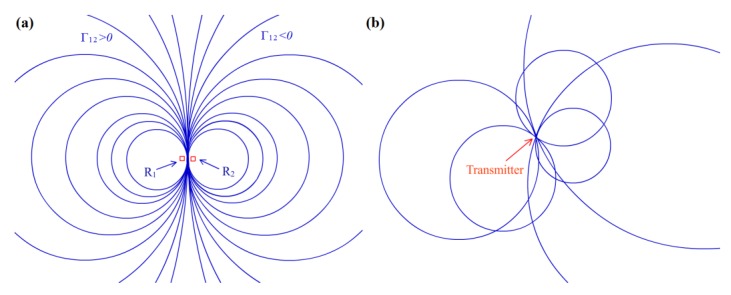
(**a**) Associated circles corresponding to the RSS differences Γ_12_. The receivers are located at R_1_ and R_2_; (**b**) Transmitter at the intersection of 6 associated circles, assuming Equation (2) is perfectly followed.

Each *θ*_12_ value corresponds to a different non-intersecting circle, and any point on the plane lies on a unique circle of the form of Equation (7) or (9). Figure 1a shows the corresponding situation and the associated circle, and when |*θ*_12_|→0, the circle become larger and converges to the perpendicular bisector of the line separating *R*_1_ and *R*_2_. Thus, the localizing problem is reduced to finding the common intersection of multiple associated circles on the plane through some optimal manner. Figure 1b shows an illustration of the transmitter location at the intersection of 6 associated circles of 4 receivers.

### 2.3. Least Squares Solution

Owing to measurement errors, multipath propagation and shadowing effects, the measured RSS deviates from Equation (2). Consequently, Equation (2) is modified to add the noise expression as shown in Equation (10).

(10) $$\bar{\Gamma }=\Gamma +\varepsilon =E-10\gamma \log_{10}(d)+\varepsilon$$

where $\bar{\Gamma }$ represents the measured RSS and *ε* denotes a zero-mean random variable with variance *σ^2^*. For *n* receivers located at *R_k_* = (*x_k_*, *y_k_*), 1 ≤ *k* ≤ *n*, the measured RSS $\bar{\Gamma }$*_k_* at *R_k_* can be defined as Equation (11).

(11) $${\bar{\Gamma }}_{k}=E-10\gamma \log_{10}(d_{k})+\varepsilon_{k},1\leq k\leq n$$

where *d_k_* represents the Euclidean distance from the transmitter to the receiver at *R_k_*, 1 ≤ *k ≤ n*, and *ε_k_*, 1 ≤ *k ≤ n*, are independent, identically distributed zero-mean random variables with variance *σ^2^*. Set ${\bar{\Gamma }}_{kl}={\bar{\Gamma }}_{k}-{\bar{\Gamma }}_{l}$, which can be calculated by Equation (12).

(12) $$\begin{array}{l} {\bar{\Gamma }}_{kl}=10\gamma \log_{10}(d_{l}/d_{k})+\varepsilon_{k}\\ =5\gamma \log_{10}\left[\frac{{\left(x-x_{l}\right)}^{2}+{\left(y-y_{l}\right)}^{2}}{{\left(x-x_{k}\right)}^{2}+{\left(y-y_{k}\right)}^{2}}\right]+\varepsilon_{kl} \end{array}$$

where (*x*, *y*) is the location of the transmitter and *ε_kl_*, 1 ≤ *k* < *l* ≤ *n*, are zero-mean random variables with a variance of 2*σ^2^*. Set Ω_n_ as Equation (13) and $\Omega \subset \Omega_{n}$ to be a subset of the set of all receiver pairs.

(13) $$\Omega_{n}=\left\{(k,l)|1\leq k<l\leq n\right\}$$

The estimation of the transmitter located at (*x*, *y*) can be obtained by minimizing the objective function *L*_Ω_(*x*, *y*) defined in Equation (14) as a nonlinear least squares (LS) problem.

(14) $$L_{\Omega }(x,y)=\sum_{(k,l)\in \Omega }{\left\{{\bar{\Omega }}_{kl}-5\gamma \log_{10}\left[\frac{{\left(x-x_{l}\right)}^{2}+{\left(y-y_{l}\right)}^{2}}{{\left(x-x_{k}\right)}^{2}+{\left(y-y_{k}\right)}^{2}}\right]\right\}}^{2}$$

Because of the complexity of implementation, the nonlinear problem of Equation (14) must be simplified for embedded systems. The closed-form LS solution proposed in [32] derives a linear system to reduce the computational cost of the algorithm. An extended total least squares (ETLS) method is introduced in [13] to transform the nonlinear formula. Moreover, a Hessian modification and adaptive weights are employed to obtain the efficient location estimate of the transmitter. However, these algorithms only improve the efficiency of the estimation procedure by transforming the nonlinear least squares question described in Equation (14); they do not consider the accuracy of the information provided by each receiver.

In practice, the RSS value will deviate from the precise model because of the complex environment. The deviations for receivers vary owing to their different locations. Figure 1b shows that the transmitter (target node) needs at least three associated circles to acquire its location by intersection. Thus, not all of the receivers (anchor nodes) are needed in the estimation; only receivers with relatively more precise RSS values are considered. To select the proper receiver candidates for the estimation of the transmitter, a confidence value can be assigned to each receiver node to indicate the accuracy of its parameters. Furthermore, an automatic calibration mechanism is needed to update these values.

## 3. Confidence Calibration

As noted above, three associated circles are sufficient for confirming a transmitter’s location. How to evaluate the accuracy of the receivers (anchor nodes) is the main question considered in this section. A set of confidence values is proposed to indicate the accuracy for each anchor node in the WSN. The anchor nodes with higher confidence values have a higher probability of being chosen for estimation. This mechanism improves the accuracy of localization in the system.

### 3.1. Confidence Construction

**Figure 2:**
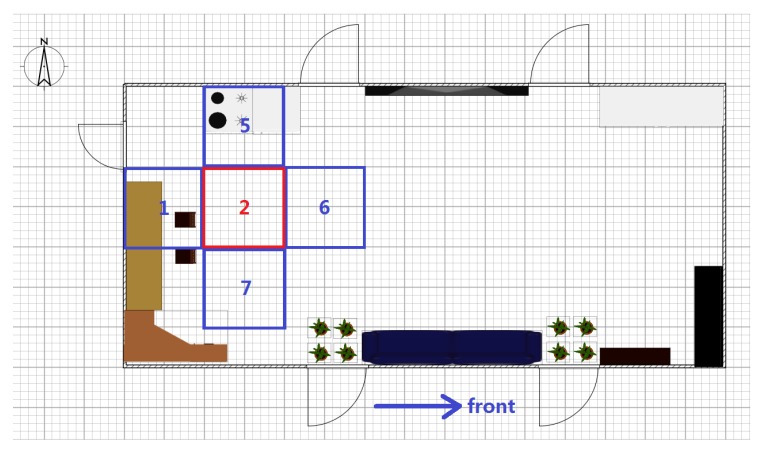
Illustration of indoor region partitioning.

**Figure 3:**
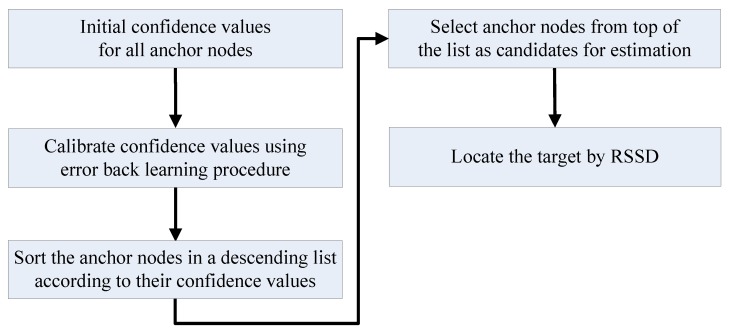
Procedure of localization by RSSD without energy control.

The confidence value of an anchor node is a metric of credibility for its RSS information in localization applications. Because the RSS value is strongly influenced by the distance between the anchor node and target, the confidence values for an anchor node should be related to the target position on the map. Hence, the indoor space for localization should first be segmented into many square partitions with numbers according to the area parameter *Sr* set by the designer, and Figure 2 shows four neighboring partitions for illustration. Then, the confidence values can be calculated for every anchor node for each partition. More partitions are generated for a smaller *Sr*, which increases the localization precision after calibration but requires more time for preparation. A table can be constructed to contain the confidence values for all anchor nodes in one certain partition. Then, the estimation is continued by choosing suitable anchor nodes as candidates. Without energy control, the anchor nodes are sorted in descending order according to their confidence values, and then, several nodes on top of the table are chosen for the estimation. The number of candidates for estimation depends on the error permission and time cost due to the balance between accuracy and computational cost. The entire procedure of localization without energy control is shown in Figure 3.

### 3.2. Back Propagation Learning

**Figure 4:**
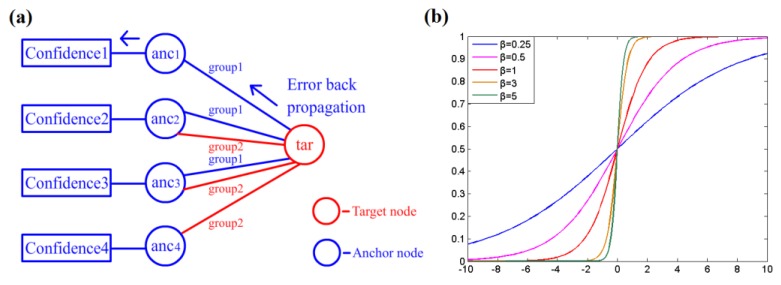
(**a**) Illustration of the back propagation learning procedure; (**b**) Curve of the back propagation function *f*(*x*).

The confidence values for each anchor node are acquired before estimation by the learning procedure. In this work, error back propagation (BP) learning is introduced to obtain the confidence values at near, middle and far distances. The BP method is often employed in BP artificial neural networks and achieves good performance. The learning procedure is performed by calibration, as shown in Figure 4a: a measuring target with a known position is deployed in the testing area, and then, its location is estimated by a group of anchor nodes using RSSD. The difference between the estimated location and actual position is calculated according to Equation (15).

(15) $$\Delta e=P_{e}-P_{r}$$

where *P_e_* represents the estimated location of the testing target and *P_r_* denotes the actual position. Then, the relevant confidence value *C_new_* can be refreshed by Equation (17), where *C_pre_* represents the previous confidence value of the anchor node obtained in the last step and *σ* is a weighting coefficient for learning speed. A large value of *σ* increases the speed of learning with more possible error, whereas a small value requires more time for learning. *ω* is a regulative constant for adjusting the influence of feedback. *f*(*x*) is a back propagation function shown in Equation (16); its return value determines the influence from the localization error. *β* is a parameter of *f*(*x*) for adjusting the shape of the curve shown in Figure 4b. A large *β* increases the sensitivity of the confidence value to localization error. After several rounds of calibration, all of the confidence values are normalized by Equation (18).

(16) $$f(x)=\frac{1}{1+e^{-\beta x}}$$

(17) $$C_{new}=C_{pre}-\sigma \cdot \omega \cdot C_{pre}\cdot (f(\left|\Delta e\right|)-0.5)$$

(18) $$C_{norm}=\frac{C_{org}-C_{\min}}{C_{\max}-C_{\min}}$$

where *C_org_* represents the original confidence value before normalization and *C_norm_* denotes the confidence value after normalization. *C_min_* is the minimum value of all confidence values, and *C_max_* is the maximum value. Thus, all of the values are mapped to a range of [0,1] by normalization.

For each partition on the map, there may be several points for calibration. At each point, a certain number of anchor nodes are grouped to estimate the target position *P_e_*, and the number was set to 3 in this work. If there are 8 anchor nodes with stable RSS signal strengths at a calibrating point, there are 56 possible combinations for choosing 3 nodes. Thus, there are 7 back propagation times for each anchor node in one calibration, and *ω* can be set to 1/7. All of the confidence values are initialized to 1 at the beginning of the calibration procedure and then decreased by error back propagation. After the calibration work at all points for this partition is completed, the values are normalized and stored in a table for further use.

### 3.3. Automatic Calibration

**Figure 5:**
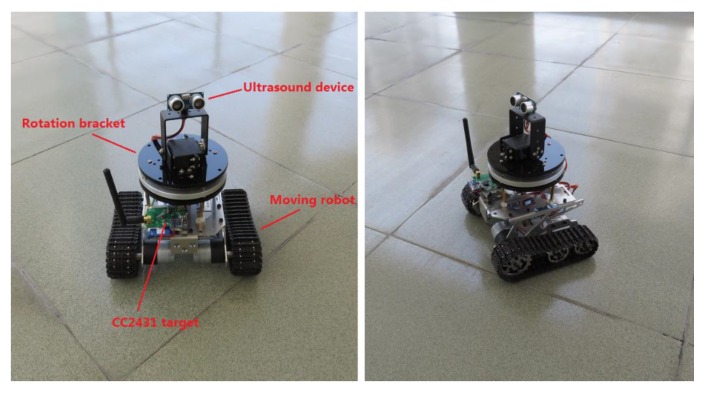
Illustration of the automatic calibration equipment.

**Figure 6:**
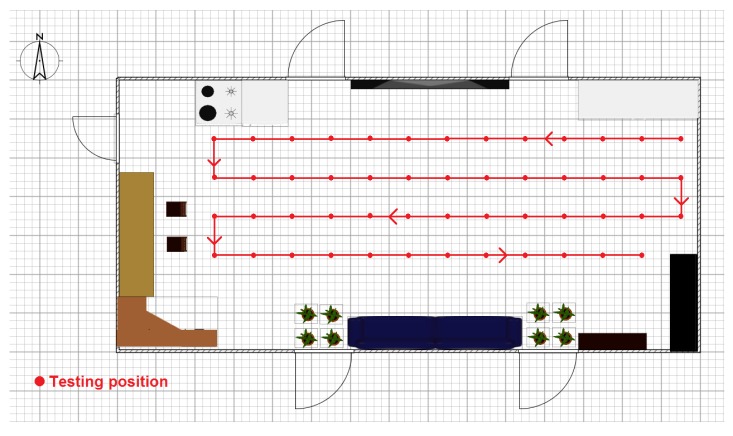
Illustration of the moving trace of the automatic calibration equipment.

Owing to the complex calibration procedure, we develop automatic calibration equipment to refresh the confidence values for each partition on the map. As shown in Figure 5, the equipment consists of a moving robot, a target device (TI CC2431, Texas Instruments, Dallas, TX, USA), a camera rotation bracket and an ultrasound device. The robot moves based on a pedrail wheel structure and two direct-current (DC) motors. The DC motor is driven by a dual full-bridge driver L298N from STMicroelectronics (Geneva, Switzerland), and its speed is regulated by the pulse width modulation (PWM) signal from a R5F100FCA MCU made by Renesas Electronics (Tokyo, Japan). Thus, the robot can change its movement direction via a speed difference between the two DC motors. The ultrasound device is equipped on the camera rotation bracket, and the bracket can rotate around the center point via a stepper motor. Hence, the ultrasound device can help to determine the robot position on the map by sampling the distance between the robot and wall in a certain direction. During the calibration procedure, the robot moves along the setting trace to calculate the confidence value for each anchor node for different area partitions, as shown in Figure 6. The robot position deviation during movement can be revised by remote instructions from the operator, and the data sampled from the ultrasound sensor can help to complete this work.

## 4. Target Localization

If an object stays at a location statically or moves slowly, its position can be detected by the RSSD method with high precision. However, when a target moves fast or suddenly changes its moving direction, the estimated position may deviate from the real position because of fluctuations in the RSS values from the anchor nodes. Moreover, the estimated value will be stabilized several seconds after the movement. It is difficult to acquire the exact position of a moving target only using RSSD. Thus, an estimation involving the history of the movement behavior is a more powerful tool for the localization of a moving target. In this work, the movement behavior of the target was described by the history of locations recorded previously and the moving parameters were sampled by a magnetometer and accelerometer. After the movement history is acquired, the UKF is employed to reduce the noise and improve the accuracy of the estimation.

### 4.1. Movement Measuring

To estimate the movement status of an object in time, an ADXL345B 3-axis accelerometer made by Analog Devices Inc. (Norwood, MA, USA) and an HMC5883L magnetometer from Honeywell (Morristown, NJ, USA) are used to acquire the moving parameters of the target. The ADXL345 is an ultralow power, 3-axis accelerometer with 13-bit resolution measurement at up to ±16 g, which is able to measure the dynamic acceleration resulting from motion or shock with a high resolution of 3.9 mg/LSB and output data through either an SPI or I^2^C digital interface. The instrument can detect inclination changes of less than 1.0°, and its extremely low power dissipation makes it a suitable choice for mobile applications.

The target position at time t only depends on its position at time t-1 and its average speed during the time interval. Thus, the position of the target can be estimated by Equation (20), where x_t_ and y_t_ represent the x and y coordinates at time t, respectively; v_x|t_ and v_y|t_ are the speed components along the *x*-axis and *y*-axis at time t, respectively; and a_x|t_ and a_y|t_ denote the acceleration components along the *x*-axis and *y*-axis at time t, respectively. a_x|t_ and a_y|t_ can be acquired by Equation (19), where a_t_ is the total acceleration vector obtained from the accelerometer, Axr denotes the angle between vector a_t_ and the *x*-axis, and Ayr denotes the angle between vector a_t_ and the *y*-axis.

(19) $$\{\begin{array}{c} a_{x|t}=a_{t}\cdot \cos(Axr)\\ a_{y|t}=a_{t}\cdot \cos(Ayr) \end{array}$$

(20) $$\{\begin{array}{c} x_{t}\approx x_{t-1}+(v_{x|t-1}+a_{x|t}\cdot \Delta t/2)\cdot \Delta t\\ y_{t}\approx y_{t-1}+(v_{y|t-1}+a_{y|t}\cdot \Delta t/2)\cdot \Delta t \end{array}$$

**Figure 7:**
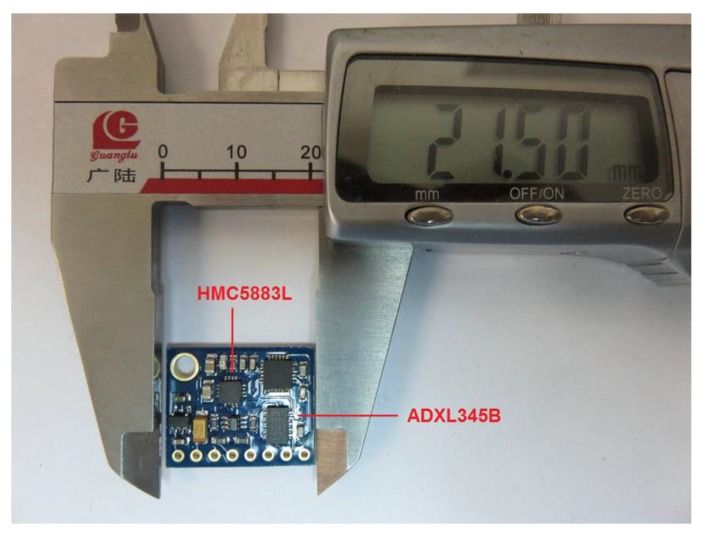
Hardware illustration of the accelerometer and magnetometer.

The Axr and Ayr angles may vary during movement owing to the rotation of the target, and a magnetometer can be used to address this problem. Magnetometers are measurement instruments used to measure the strength and direction of a magnetic field at a point in space. In this application, the magnetometers are used as compasses to detect the angle between the target and *x*-axis. The HMC5883L is a surface-mount compass IC for low-field magnetic sensing, such as compassing and magnetometry. The sensor is constructed with low cross-axis sensitivity to measure Earth's magnetic fields with a range of ±8 gauss and a resolution of 5 milli-gauss. These two chips have been integrated on a small PCB board as a wearable module, as shown in Figure 7. Thus, the moving parameters of the target can be acquired in real time.

### 4.2. Unscented Kalman Filter

Owing to the measurement noise from real-time devices, the estimated position of the target may deviate from its real location. Thus, the Kalman filter can be used to improve the accuracy of localization by obtaining the optimal estimation of disturbance by a series of measurement and process noise. However, the classical Kalman filter only achieves a good performance in linear systems. Therefore, the extended Kalman filter (EKF) and UKF are proposed. The EKF algorithm achieves a good effect in the nonlinear system by performing linearization in a nonlinear model. However, the linearization error gradually accumulates with an increasing number of iterations and finally reduces the accuracy of the estimation. Moreover, the calculation of two Jacobian matrices in the EKF procedure burdens the processor. The UKF is an improved nonlinear algorithm that uses the unscented transformation (UT) instead of linearization for calculating the state predictions and covariances [33]. The UKF estimation procedure is shown in Algorithm 1 and includes two main steps: prediction and update. The estimated location of a target is viewed as the observation of the UKF.

**Algorithm 1.** Procedure for the unscented Kalman filter.
**Step 1. Prediction**Step 1.1 InitializationThe model of the UKF for a nonlinear system is generally expressed as follows:    x(k)=f[x(k−1),u(k)]+w(k)    z(k)=h[x(k)]+v(k)where *f*(*x*) represents the predicted function, *x*(*k*) denotes the state vector, *u*(*k*) is the input vector, and *w*(*k*) is the Gaussian process noise, with a covariance of *Q*(*k*). Moreover, *h*(*x*) is the measurement function, and *v*(*k*) represents the Gaussian measurement noise, with a covariance of *R*(*k*). Furthermore, *w*(*k*) and *v*(*k*) are all considered as zero-mean Gaussian noise.At the first step, the state vector *x*(*k*) is initialized with a mean of *x*_0_, and the error covariance *P*_0_ is assigned as follows:$\;{\bar{x}}_{0}=E[x_{0}]$$\;P_{0}=E[(x_{0}-{\bar{x}}_{0}){\left(x_{0}-{\bar{x}}_{0}\right)}^{T}]$
Step 1.2 Computing sigma pointsAccording to the mean ${\bar{x}}_{k-1}$ and error covariance *P*_*k*−1_, a set containing 2n+1 weighted samples, which are called sigma points, are computed according to the following equation. The *n* in the formulas refers to the dimension of the state vector.$\;X_{k-1}^{\left(s\right)}=\{\begin{array}{ll} {\bar{x}}_{k-1}, & s=0\\ {\bar{x}}_{k-1}+{\left(\sqrt{\left(n+\lambda \right)}P_{k-1}\right)}_{s}, & s=1,\ldots n\\ {\bar{x}}_{k-1}-{\left(\sqrt{\left(n+\lambda \right)}P_{k-1}\right)}_{s}, & s=n+1,\ldots 2n \end{array}$
where *λ* is a scaling factor that can be acquired as follows:$\;\lambda =\alpha^{2}(n+t)-n$where *α* is a small positive constant. *t* is the secondary scaling factor, which is set to 0 in our work. Thus, the mean weights *W_m_* can be defined as follows:$\;W_{m}^{\left(s\right)}=\{\begin{array}{ll} \frac{\lambda }{\lambda +n}, & s=0\\ \frac{\lambda }{2(\lambda +n)}, & s=1,\ldots 2n \end{array}$
Step 1.3 Propagating sigma pointsThe sigma points are propagated in the predicted function *f*(*x*).
$\;X_{k|k-1}^{\left(s\right)}=f(X_{k-1}^{\left(s\right)},u_{k-1})$
Step 1.4 Calculate the prior state and error covarianceThe prior state estimate can be calculated as follows:$\;{\bar{x}}_{k|k-1}=\sum_{i=0}^{2n}W_{m}^{\left(s\right)}X_{k|k-1}^{\left(s\right)}$
To obtain the prior error covariance, the variance weights *W_c_* should be acquired as follows:
$\;W_{c}^{\left(s\right)}=\{\begin{array}{ll} \frac{\lambda }{\lambda +n}+(1-\alpha^{2}+\rho ), & s=0\\ \frac{\lambda }{2(\lambda +n)}, & s=1,\ldots 2n \end{array}$where *ρ* represents a parameter in the state estimate, which is generally set to 2 to obtain the optimal estimation in Gaussian distributions. Moreover, the prior error covariance can be obtained as follows:
$\;P_{k|k-1}=\sum_{i=0}^{2n}W_{c}^{\left(s\right)}(X_{k|k-1}^{\left(s\right)}-{\bar{x}}_{k|k-1}){\left(X_{k|k-1}^{\left(s\right)}-{\bar{x}}_{k|k-1}\right)}^{T}+Q$
Step 2. UpdateStep 2.1 Computing sigma pointsAfter the predicting process, a new set of sigma points is acquired. The new sigma points are calculated by the prior state estimate as follows:$\;X_{k|k-1}^{\left(s\right)}=\{\begin{array}{ll} {\bar{x}}_{k|k-1}, & s=0\\ {\bar{x}}_{k|k-1}+{\left(\sqrt{\left(n+\lambda \right)}P_{k|k-1}\right)}_{s}, & s=1,\ldots n\\ {\bar{x}}_{k|k-1}-{\left(\sqrt{\left(n+\lambda \right)}P_{k|k-1}\right)}_{s}, & s=n+1,\ldots 2n \end{array}$
Step 2.2 Calculating the predicted outputEach sigma point in the measurement function *h*(*x*) is calculated to obtain the output, which is presented as below:$\;Y_{k|k-1}^{\left(s\right)}=h(X_{k|k-1}^{\left(s\right)},u_{k})$
Furthermore, the predicted output can be acquired as follows:
$\;{\bar{Y}}_{k|k-1}=\sum_{i=0}^{2n}W_{m}^{\left(s\right)}Y_{k|k-1}^{\left(s\right)}$
Step 2.3 Computing the Kalman gain *K_k_*The predicted output covariance matrix $P_{y_{k}y_{k}}$ and cross-covariance $P_{x_{k}y_{k}}$ are necessary for obtaining the Kalman gain *K_k_*. These values can be acquired according to the following equations.$\;P_{y_{k}y_{k}}=\sum_{i=0}^{2n}W_{c}^{\left(s\right)}(Y_{k|k-1}^{\left(s\right)}-{\bar{Y}}_{k|k-1}){\left(Y_{k|k-1}^{\left(s\right)}-{\bar{Y}}_{k|k-1}\right)}^{T}+R$$\;P_{x_{k}y_{k}}=\sum_{i=0}^{2n}W_{c}^{\left(s\right)}(X_{k|k-1}^{\left(s\right)}-{\bar{x}}_{k|k-1}){\left(Y_{k|k-1}^{\left(s\right)}-{\bar{Y}}_{k|k-1}\right)}^{T}$
$\;K_{k}=P_{x_{k}y_{k}}P_{y_{k}y_{k}}^{-1}$Step 2.4 Calculate the posterior state and error covarianceAccording to the measurement information, the posterior state and error covariance estimate can be acquired by the following equations.$\;{\bar{x}}_{k}={\bar{x}}_{k|k-1}+K_{k}(Z_{k}-{\bar{Y}}_{k|k-1})$$\;P_{k}=P_{k|k-1}-K_{k}P_{y_{k}y_{k}}K_{k}^{T}$
where *Z_k_* refers to the measurement sampled from the sensors at step *k*. Moreover, the posterior error covariance is essential for the calculation in the next step.

**Figure 8:**
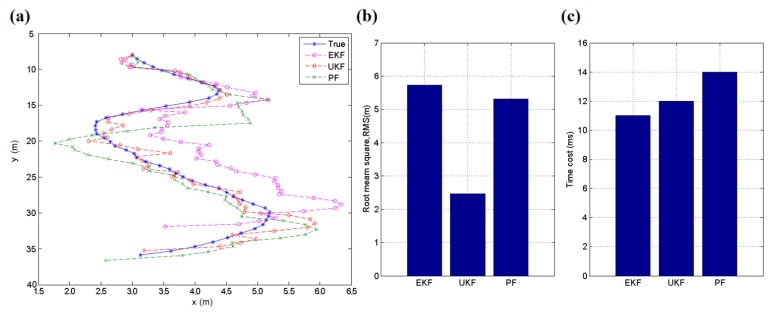
(**a**) Tracking trace of the simulation; (**b**) Root mean square (RMS) errors of the three methods during the simulation; (**c**) Time cost of the three methods during the simulation.

A simulation is performed to compare the effect in a tracking case for the EKF, UKF and particle filter (PF) [34], which is also often used in tracking applications. The simulation works on a 50-point dataset from a real application, and the object moves along an approximate tangent curve. There are 100 particles participating in the computation of the PF method, and measuring errors are included in the random equation. The testing results are shown in Figure 8. The UKF acquires the least root mean square error (RMSE) of these three methods in this case, and its time cost is also acceptable. If the number of particles in PF increases, the method may perform better but require more time for calculation. The UKF does not always obtain the best results but has a high probability of performing well. Owing to its relatively less adjustable parameters and computational cost, the UKF is introduced in this work to reduce the influence of measurement noise in the localization, particularly for a moving target.

### 4.3. Localization Procedure

**Figure 9:**
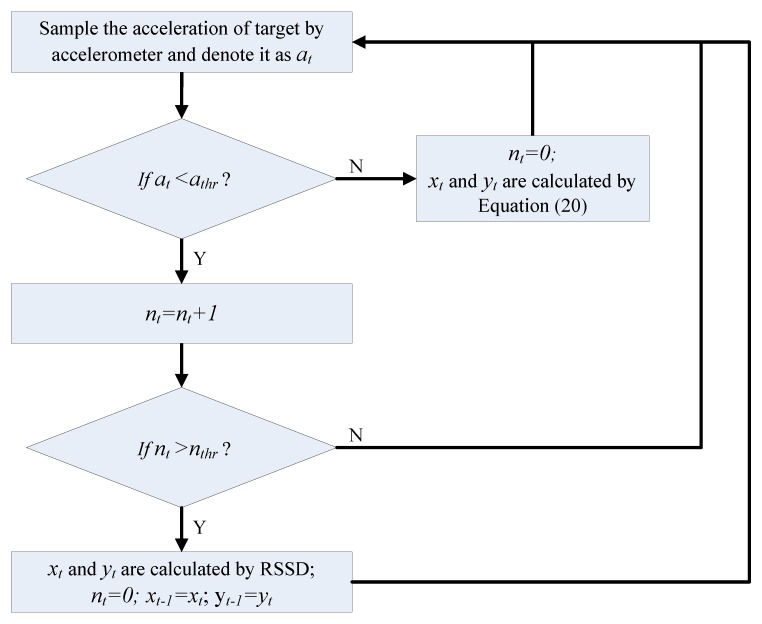
Target localization procedure.

According to the estimated position calculated by RSSD and the moving parameters obtained by sensors, the target’s movement can be tracked using simple geometrical computation. The entire target localization procedure is described in Figure 9, where a_t_ represents the acceleration of the target at time t, a_thr_ denotes a threshold for the acceleration a_t_, n_t_ is the time interval count for stability, n_thr_ is the threshold for the count n_t_, and x_t_ and y_t_ are the coordinates of the target at *x*-axis and *y*-axis at time t, respectively. When a_t_ ≥ a_thr_, the target is considered to be moving, and its position is estimated by Equation (20) according to its position at time t − 1 and the parameters obtained by the sensors. If a_t_ < a_thr_, the position of the target is nearly static and must be confirmed by several additional time intervals indicated by n_thr_. When the static status is verified, its location can be estimated by the RSSD method introduced in this work. The threshold parameters a_thr_ and n_thr_ can influence the effect of the estimation. A large a_thr_ value or small n_thr_ value can tolerate more noise from the measurement for the system with lower accuracy. Furthermore, a large n_thr_ value reduces the reaction speed of the system. Thus, suitable values for a_thr_ and n_thr_ are important factors for this localization and should be chosen according to the application environment and sensor parameters of the hardware. In our experiments below, a_thr_ is set to 0.3 m/s^2^ and n_thr_ is set to 5.

## 5. Anchor Selection and Energy Control

As noted above, the target requires at least three RSSD circles to estimate its location. Moreover, the RSS values from three anchor nodes are sufficient to build three RSSD circles for estimation. The additional anchor nodes may improve the localization accuracy but lead to a greater computational burden on the system and increased power consumption by the anchor nodes. The localization application in this work is mainly based on WSNs, and the advantage depends on its mobile anchor nodes and variable topological structure. The absence of a need to fix the power supply allows it to be deployed rapidly in extreme environments, such as coal mines, wild fields or mountain camps. However, for indoor conditions, the devices are restricted to acquire energy from nature, and the power must come from batteries equipped on the sensor nodes. Thus, energy control is considered a critical issue in real environments.

### 5.1. Structure for Energy Control

**Figure 10:**
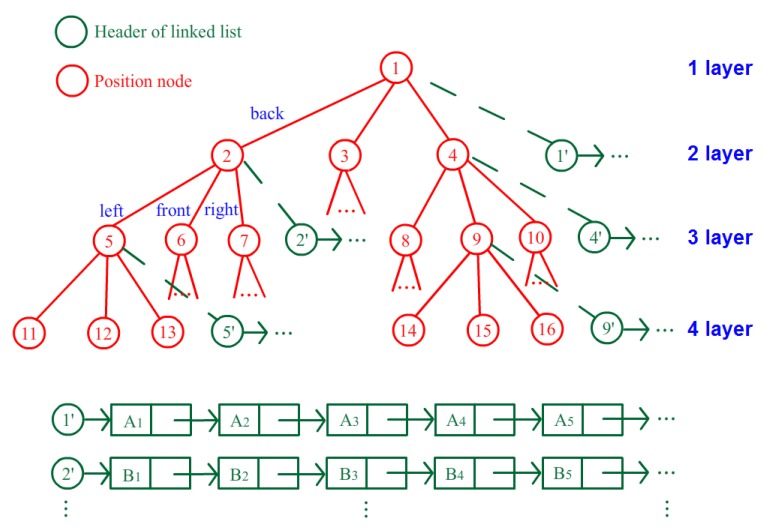
Illustration of the quadtree structure.

Only the anchor nodes involved in the estimation need to be activated; other nodes can be set to sleep to save energy. However, the waking procedure of a sleeping node requires several hundreds of milliseconds, which may lead to discontinuous estimation process during target movement. Thus, the anchor nodes associated with the neighboring region of the target must be activated for preparation. Because the positions of the target at time t − 1 and time t are correlated, a quadtree structure, as shown in Figure 10, is introduced to help select the awakened anchor nodes. The indoor space needs to be segmented into several square partitions with numbers according to the parameter Sr, and Figure 2 shows four neighboring partitions in that example. Then, the region correlation between neighboring partitions can be described as the relationship between the parent and children of the tree. Figure 10 shows an illustration of the tree structure, and each element of the tree represents an area partition. The parent of a tree node associates with its back partition, and its three children, as marked by the red circle in Figure 10, denote the left, front and right regions. Figure 2 shows the four neighboring partitions 1, 5, 6, and 7 surrounding partition 2, and they are described in the quadtree of Figure 10. After the construction of the quadtree for the entire space, the structure reveals the region correlation of this space, and the necessary anchor nodes can be activated according to the elements of the tree.

Every node of the quadtree has its fourth child marked by a green circle in Figure 10, which is a header of the linked list of anchor node candidates for this partition. The linked list is organized as a queue according to the suitable degree of these anchor nodes for localization in the partition. Thus, the selection can be made using the top elements of the list. Moreover, the awakened anchor nodes can be selected by the structure of the quadtree. The current element that represents the target standing partition is first found out on the quadtree. Then, the anchor nodes associating with elements several layers up or down the current element on the quadtree are activated for preparation. Therefore, only anchor nodes associated with the surrounding partitions of the target use energy to wait for the possible entrance of the target, and the other nodes can fall asleep to save energy. A level constraint parameter Lr is set to indicate the range of activated elements on the tree. If Lr = 2, the anchor nodes associated with the elements two layers up and down from the current element (including the current layer, five layers in total) need to be activated. A large Lr will improve the response speed and continuity of localization but will have a high energy cost.

### 5.2. Anchor Node Selection

The second problem is to improve the localization accuracy as much as possible under energy control. Because the RSS values are influenced by the circumstances surrounding the anchor nodes, the localization accuracy is related to the selection of anchor nodes in the estimation. How to choose the best anchor nodes for estimation under the setting of a fixed number Tr of anchor nodes used at each time of estimation is an optimization problem to achieve a balance between accuracy and energy consumption. To determine the best decision, the selection score is calculated for each anchor node to indicate its fitness as a candidate for estimation as follows:

(21) $$E_{norm}=\frac{E_{now}-E_{\min}}{E_{full}-E_{\min}}$$

*N_Score_* = *φ·C_norm_* + *η·E_norm_* (22)

where *E_full_* represents the full level of the battery equipped on the anchor node, *E_min_* is the minimum level of the battery for maintaining the necessary work of the anchor node, *E_now_* is the current level of the battery on the anchor node, *E_norm_* denotes the energy level after normalization, which has the range of [0,1], *C_norm_* represents the confidence value for the anchor node after normalization, *N_Score_* is the selection score for the anchor node, which indicates its possibility of being chosen for estimation considering the energy situation, and *φ* and *η* are scaling factors for regulating the ratio of the confidence value and energy level in the calculation of the selection score.

After the definition of the selection score, for each area partition (an element on the quadtree), the selection score is calculated for each relevant anchor node, and all of the anchor nodes are sorted as a linked list in descending order. The header of the linked list is assigned to the fourth child of the quadtree node, as shown by the green circle in Figure 10. The three or more elements on the top of the linked list can be selected as the candidates for estimation. Furthermore, the linked list should be refreshed after a fixed time interval to reflect the change in the energy levels of the anchor nodes over time. Because of the large difference values between the top and bottom elements of the linked list, there is no need to add all of the anchor nodes to the linked list. A truncated parameter *Cr* can be set to allow only a fixed number of anchor nodes with high selection scores to join the list because the nodes with low scores have a low probability of participating in the estimation. In our experiments below, *Cr* is set to 8 nodes.

## 6. Implementation and Experiment

**Figure 11:**
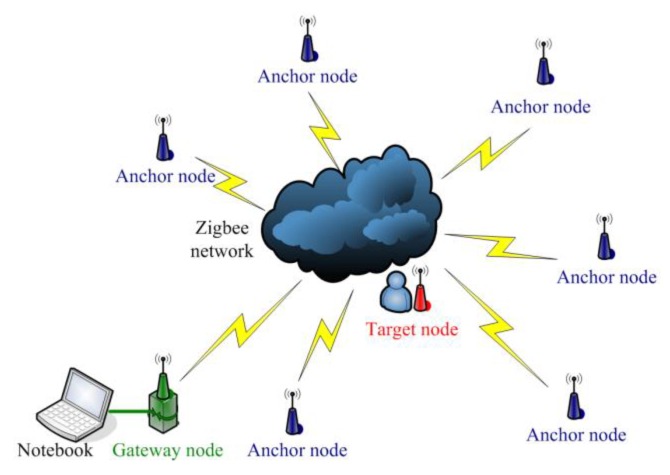
Illustration of the structure for the WSN localization system.

**Figure 12:**
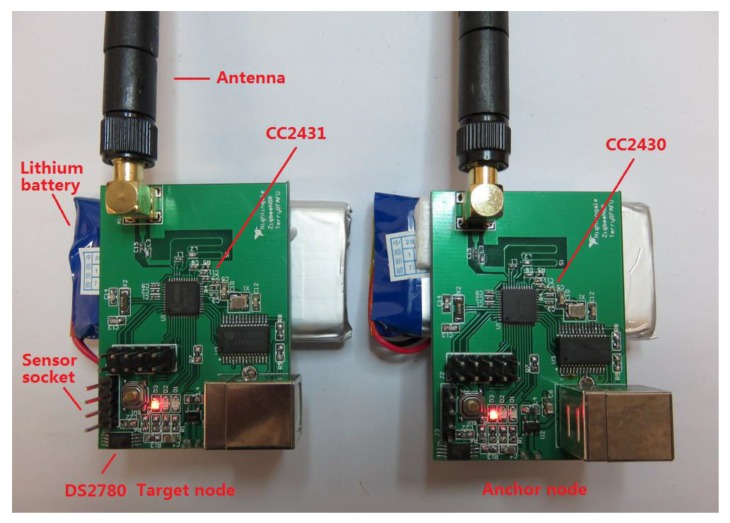
Illustration of the anchor node and target node.

The WSN for indoor localization application is implemented on the TI CC2430 and CC2431 platforms. The CC2430 combines an excellent RF transceiver with an industry-standard enhanced 8051 MCU. The system is highly suited for ultra-low power consumption. The CC2431 is a true System-On-Chip (SOC) for ZigBee (IEEE 802.15.4) solutions. The chip includes a location detection hardware module that can be used to measure the signal strength from the anchor nodes. Based on this measurement, the location engine can calculate an estimate of a blind node’s position. CC2431 and the CC2430 are pin compatible, and the MCU and RF parts of CC2430-F128 are identical to CC2431, except the location engine. According to the structures of these two chips, the localization system is built using three types of sensor nodes: anchor nodes, target nodes and gateway nodes. The anchor nodes with known location provide geographical information for estimation, and the target node can find its location on the map by estimating the difference of position between it and the anchor node. The gateway node is used to organize the communication of the WSN. The results are then presented by application software on a notebook, and the structure of the system is illustrated in Figure 11. The hardware illustration of the sensor nodes is presented in Figure 12. Furthermore, a Maxim DS2780 chip is used to measure the residual energy level of the battery on the nodes, which is a 16-bit professional measure IC for estimating the available capacity for rechargeable lithium batteries.

**Figure 13:**
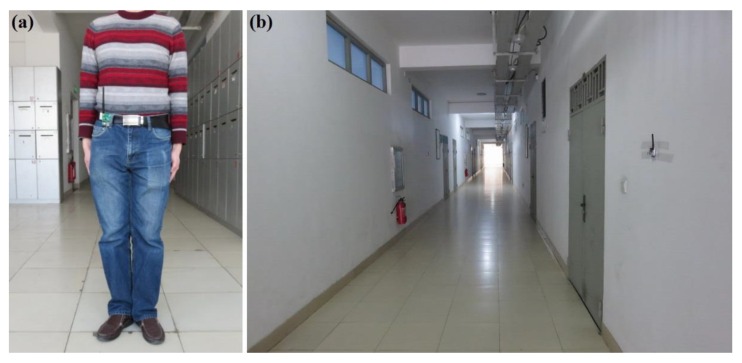
(**a**) Illustration of the blind target; (**b**) Illustration of testing scenario 1.

**Figure 14:**
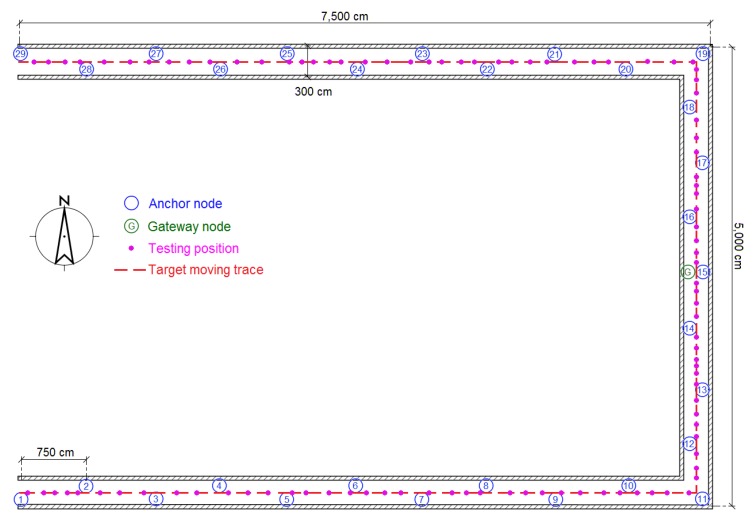
Map and nodes’ deployment for testing scenario 1.

The experiments were performed in three typical scenarios to test the proposed methods. The first scenario was a narrow corridor in a teaching building, as shown in Figure 13b, for simulating the environment of a narrow pit. Figure 14 shows the map of this scene, and 29 anchor nodes were deployed with an interval of 7.5 m to localize the blind target by RSSD. The blind target can be carried in two ways: by a man, as shown in Figure 13a, or equipped on a robot, as shown in Figure 5. The accuracy of RSS information provided by an anchor node is mainly influenced by its surrounding environment, such as, metal, special building structure or other radio emitter. Thus, the calibration procedure in the paper is aim to figure out the ranking of anchor nodes with consideration of their accuracy for localization. During the whole calibration procedure, the same robot with the target device (CC2431) several centimeters up ground is used and it is found that the same devices’ height has the same influence on all the anchor nodes. Therefore, we get the result that devices’ height could not affect the ranking of anchor nodes in the procedure. For testing, the target node may be carried by a man and therefore the devices’ height may be different from the height of calibration robot after calibration. The positions of all the anchor nodes are kept the same from calibration to testing procedure and their surrounding environment is not changed, so the different heights of devices carried by tester would just have influences on the average position RMSE (root mean squared error) of localization system. However, it is not caused by calibration procedure, as the ranking of anchor nodes is not changed. As shown in Figure 14, 100 testing positions were set along the moving trace marked by the red dotted line to test the localization ability of the system in the entire area. Under scenario 1, the moving trace was set in the middle of the corridor, and the testing positions were selected by random spacing distances. Owing to the fluctuation of the RSSI signal during target movement, there are two different testing modes in our experiments: (1) still mode: localization is operated 10 s after the blind target stops at the testing position; (2) moving mode: the target node is equipped on a robot and moved along the moving trace, localization is performed during the entire time and the estimated values at the testing positions are determined to evaluate the performance of the system.

In the experiment of scenario 1, each partition is defined as a 2 × 2 m^2^ area (Sr = 2), and the level constraint parameter Lr is set to 2. The anchor nodes are set up on a wall at a height of 1.6 m ± 0.1 m, and four anchor nodes (Tr = 4) are selected for estimation at each time of localization. In the BP learning procedure, the speed coefficient σ is set to 0.3 and β is set to 0.5 for Equations (16) and (17). The confidence values for all partitions are fully calibrated by a set of testing points with a distance interval of 0.5 m on the map. To balance the precision and energy cost, φ is set to 0.7 and η is set to 0.3 for Equation (21). For the still mode, a man moves the target node and the localization function is tested 10 s after he stands still. For the moving mode, a robot moving at a speed of 3.6 km/h carries the blind target. The experiments were performed 10 times on each testing position to get average results, and the UKF tool was employed by all methods for the moving target.

**Figure 15:**
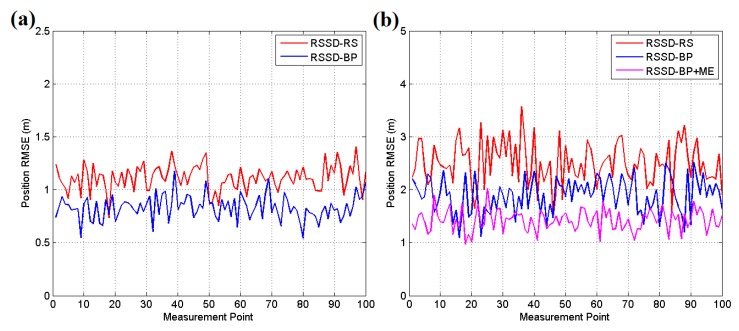
(**a**) RMSE of the still target for each testing position in scene 1; (**b**) RMSE of the moving target for each testing position in scenario 1.

**Figure 16:**
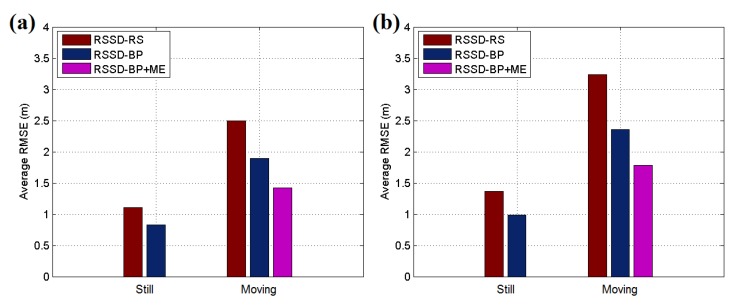
(**a**) Average RMSE of the still and moving targets in testing scenario 1; (**b**) Average RMSE of still and moving targets in testing scenario 2.

Figure 15 and Figure 16a present the experimental results for each testing position in scenario 1, and the localization accuracy is measured by the average position root mean squared error (RMSE). According to the datasheet of CC2431, the chip contains a location engine with readout resolution of 0.25 m. However, in actual application, the localization system is very hard to achieve this ideal accuracy due to the obstacles or noise in environment. The RSSD-RS method, which chooses the anchor nodes for its estimation by random selection, achieves an average position RMSE of 1.11 m in the still testing mode, and the RSSD-BP method, which chooses anchor nodes by proposed confidence values and the BP learning procedure, acquires an average RMSE of 0.83 m in the still mode. In the moving testing mode, the RSSD-BP has an average RMSE of 1.89 m, which is 75.6% of that of RSSD-RS, as shown in Figure 16a. The movement behavior of an object may lead to a large increment in the localization error because of the instability of the RSSI signal during activity. However, movement estimation based on the accelerometer and magnetometer introduced in Section 4.1 can help to achieve higher accuracy in the moving environment. Thus, the RSSD-BP+ME group, which uses the RSSD-BP with movement estimation, obtains the lowest average RMSE of 1.42 m in Figure 16a owing to its employment of movement information instead of RSSI data for estimation during activity. Furthermore, Figure 15b shows that the RSSD-BP+ME method can reduce the fluctuations in the RMSE curve for more homogeneous results for different testing positions.

**Figure 17:**
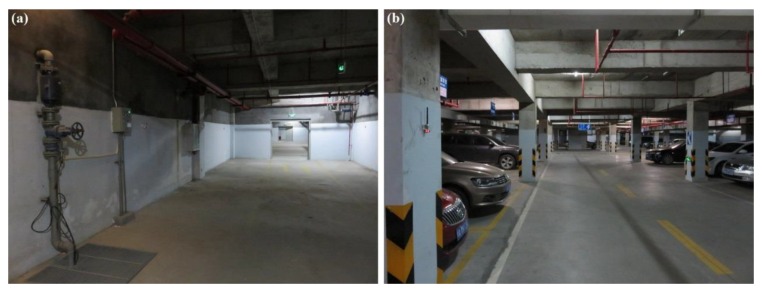
(**a**) Illustration of testing scenario 2; (**b**) Illustration of testing scenario 3.

**Figure 18:**
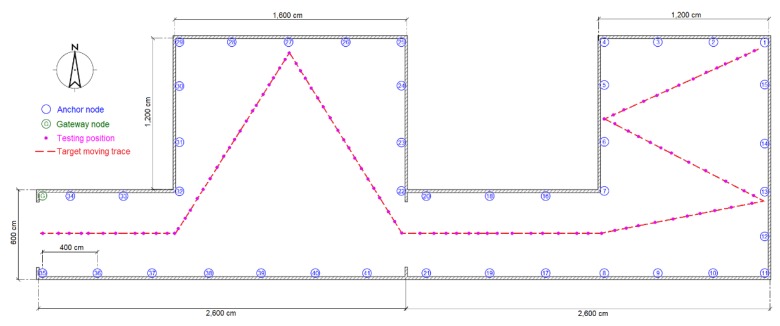
Map and nodes’ deployment for testing scenario 2.

Because WSN localization systems are often deployed in environments without GPS satellite signals, the second and third testing scenarios are in a basement to simulate the circumstances. Figure 17a shows an illustration of testing scenario 2, which is a pump and electrical room in the basement of a building underground without GPS or mobile phone signals. The metal equipment in the basement produces disturbances in the RSSI signal, which can be used to test the robustness of the system. Figure 18 shows the map and nodes’ deployment of scenario 2, and the interval between two neighboring anchor nodes is set to 4 m. The 100 testing positions are arranged on a set of broken lines, and the running parameters of the system are set to be the same as in scenario 1. The UKF is employed to increase the accuracy for locating the moving target, and the testing results are shown in Figure 16b. In the still testing mode, the RSSD-BP had an average RMSE of 0.99 m, which is 72.3% of the 1.37 m of RSSD-RS. Thus, the BP learning procedure increases the accuracy by selecting suitable anchor nodes for estimation. Moreover, the RSSD-BP+ME also acquires the highest accuracy in the moving test.

**Figure 19:**
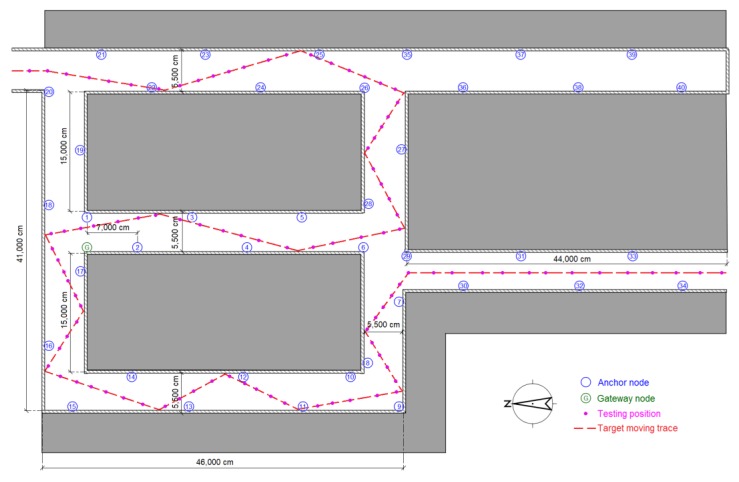
Map and nodes’ deployment for testing scenario 3.

**Figure 20:**
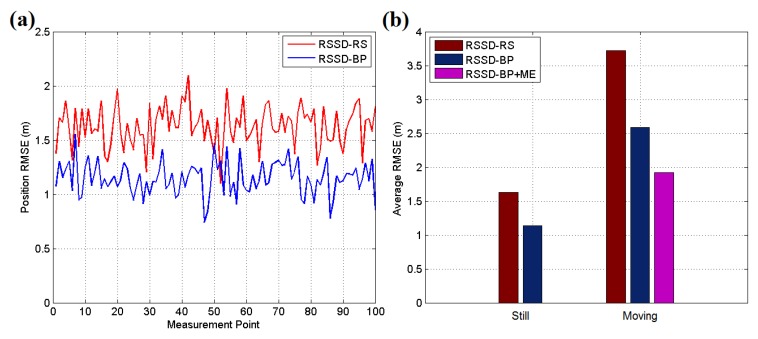
(**a**) RMSE of the still target for each testing position in scenario 3; (**b**) Average RMSE of still and moving targets in testing scenario 3.

An underground garage is selected as the third testing scenario to evaluate the proposed algorithms as shown in Figure 17b. The garage environment is sufficiently complex for testing the robustness of the proposed methods and is a common application environment in daily life. Figure 19 shows the map and nodes’ deployment of testing scenario 3, and the interval of nodes is set to 7 m at most locations in the scenario. The system parameters are set to be the same as in the two previous scenes, except *σ* = 0.2, and the UKF is used to reduce the estimation noise for the moving target. The testing results are presented in Figure 20. The RSSD-BP method has an average RMSE of 1.15, which is 29.4% lower than the 1.63 m of the RSSD-RS method in the still testing mode. Thus, in a complex environment, the BP learning procedure has advantages for localization owing to the exclusion of anchor nodes with a high estimation error. In the moving testing mode, the RSSD-BP+ME method has an average RMSE of 1.92 m, which is 48.4% lower than the 3.72 m of RSSD-RS because of the BP learning procedure and movement estimation mechanism. However, the price paid for improving the localization accuracy is the time cost for BP calibration before system running and the increased hardware required for movement estimation.

**Figure 21:**
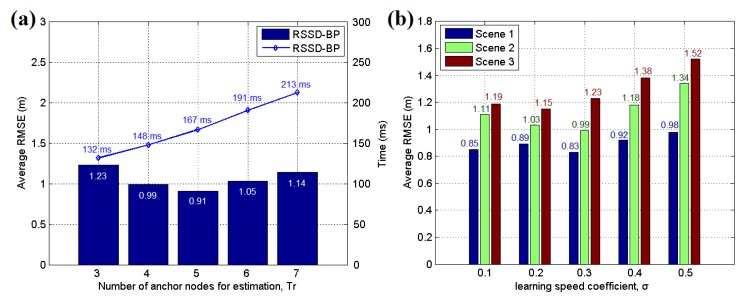
(**a**) Average RMSE of the still target estimated by different numbers of anchor nodes in testing scenario 2; (**b**) Average RMSE of the still target estimated by different learning speed coefficients in testing scenario 2.

As noted above, at least three anchor nodes are necessary for target position estimation. Furthermore, more anchor nodes can be included in the estimation through the least squares solution to improve the accuracy of the localization. To investigate the influence of the different anchor nodes in localization, a comparative test is performed in the three scenarios using a still target. The results are shown in Table 1 and Figure 21a. It can be found that the lowest average RMSE can be gotten by using four anchor nodes in scenario 1. However, in scenario 3, five anchor nodes are helpful for maintaining the best localization accuracy. Furthermore, Figure 21a show that five anchor nodes acquire the lowest average RMSE of 0.91 m with 167 ms for each time in scenario 2. However, seven anchor nodes obtain a higher value of 1.14 m. Thus, a larger number of anchor nodes does not always increase the accuracy and will increase the time cost of the estimation. The best chosen number of anchor nodes is influenced by many complex factors and the noise in environment is an important one. Besides this, the metal, building structure and obstacles in environment are also the factors. Nevertheless, we’re sure that the little increase of anchor nodes’ number would help the system to be on better performance when facing more complicated environment. For instance, in the scenario 3, many metal cars could disturb the signal of system. Therefore, the number parameter should be determined according to the real application environment to achieve a balance between accuracy and time consumption. Based on our experiments, we have found that the suitable number of anchor node for localization application is four to six under most scenarios. The learning speed coefficient *σ* in Equation (17) is also an important parameter for the BP learning mechanism in the RSSD-BP method. A large *σ* decreases the learning time but results in a higher possible error. Thus, a lower value of *σ* with more BP propagation may be suitable for complex environments. A comparative experiment was performed for these three scenarios, and the result is presented in Figure 21b. The best values of *σ* for these three scenarios are different owing to the different noise levels. However, the low *σ* values (0.1 to 0.3) are a suitable range for typical applications.

**Figure 22:**
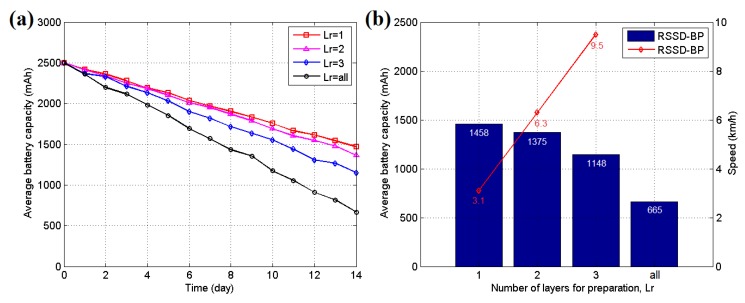
(**a**) Average battery capacity curve for different awakened parameters in testing scenario 2; (**b**) Final average battery capacity and target speed restriction for different awakened parameters in testing scenario 2.

Energy control is another key point in WSN localization systems without a fixed power supply. In our system, the anchor and target nodes are powered by LP903158 lithium batteries manufactured by the ZONCELL Corporation (Shenzhen, China) with a storage capacity of 2500 mAh. The quadtree structure proposed in Section 5, which is constructed according to the correlation between neighboring area partitions, is a method of saving energy. After the quadtree is built, several layers of anchor nodes are activated for localization, whereas the others are set to sleep to save energy. Determining the number of layers necessary to be awakened is an optimization problem. A 14-day experiment is employed to determine the answer in testing scenario 2 because its hidden position is difficult to disturb. A robot equipped with the target node is set to move back and forth automatically along the moving trace at a speed of 1 km/h during the entire time. The experiment is performed 5 times, and the average results are presented in Figure 22. Figure 22a shows the curve of the average battery capacity for all anchor nodes during the experiment. A lower number of awakened layers of nodes require less battery energy in the experiment. However, if the awakened nodes are not sufficient, the localization function may be interrupted when the target moves through the border of two neighboring partitions. Figure 22b shows the relationship between the final energy levels after the 14-day experiment and the speed restriction for each parameter in our system. If *Lr* = 1, the system has an average remaining energy of 1458 mAh, thus using 43.2% less energy than the *Lr* = all situation. However, only the localization function of the moving target with a speed of 3.1 km/h can be supported. Thus, the *Lr* value should be selected according to the speed of the target in real applications. Generally, *Lr* = 2 or *Lr* = 3 is sufficient for most applications.

## 7. Conclusions

The RSSD method allows the target to be located without knowing its transmission parameters. Within this method, only three anchor nodes are necessary to determine the position of a target. How to choose the best anchor nodes for estimation becomes an optimization problem for the system. Thus, confidence values are proposed to help in the selection of anchor nodes, and the back propagation learning procedure is introduced to calibrate the values. Energy control is another important point for WSN-based indoor localization applications. A quadtree structure is introduced to describe the region correlation between area partitions. Using the quadtree structure, the sleeping anchor nodes can be determined to save energy. A real-time system was implemented on TI CC2430 and CC2431 platforms, and the experimental results demonstrate the improvement that can be gained using these algorithms.

## Figures and Tables

**Table 1:** Average RMSE of the still target estimated by different numbers of anchor nodes in the three testing scenarios.

Scenario	Average RMSE (m)				
	*Tr* = 3	*Tr =* 4	*Tr* = 5	*Tr* = 6	*Tr =* 7
Scenario 1	1.10	0.83	0.87	0.92	1.01
Scenario 2	1.23	0.99	0.91	1.05	1.14
Scenario 3	1.44	1.15	1.06	1.23	1.33
